# Antidiabetic Potential of *Silybum marianum* (L.) Gaertn. and *Brachylaena discolor* DC (Asteraceae) in the Management of Type 2 Diabetes Mellitus

**DOI:** 10.3390/plants14213267

**Published:** 2025-10-26

**Authors:** Emmanuel A. Ayeni, Anthony J. Afolayan

**Affiliations:** Department of Biotechnology and Biological Science, University of Fort Hare, Alice 5700, South Africa; ayeniemmanuel91@yahoo.com

**Keywords:** *Silybum marianum*, *Brachylaena discolor*, diabetes, medicinal plants, Asteraceae

## Abstract

*Silybum marianum* and *Brachylaena discolor* were identified from the ethnobotanical pool of medicinal plants used in managing type 2 diabetes mellitus in South Africa. These two plants were selected based on their strength of evidence from our preliminary investigation and frequency of ethnomedicinal use. An extensive literature review was performed using major scientific databases. *Silybum marianum* and *Brachylaena discolor* have shown potential activity in decreasing blood glucose levels. Previously isolated chemical compounds validated their anti-diabetic properties, thus confirming their importance and utilization from an ethnobotanical perspective for drug discovery in the development of type 2 diabetes drugs. The traditional use of *Brachylaena discolor* involved infusion and decoction methods, and the isolation of target-based compounds will be required for anti-diabetic activities. However, the existing toxicity profile remains insufficient, especially for *B. discolor*. The findings suggest that these plants would be beneficial to the populace as an add-on dietary vegetable in lowering blood sugar levels for the treatment of type 2 diabetes. Further comprehensive studies are needed to fully establish their safety profile, particularly with long-term use or when consumed.

## 1. Introduction

Type 2 diabetes (T2DM) is a metabolic condition characterized by insulin resistance and hyperglycaemia [1]. It involves impairment of carbohydrate metabolism as a result of insufficient insulin production or a decrease in the responsiveness of peripheral tissues to insulin signaling [2]. Type 2 diabetes mellitus is a global burden disease that affects approximately 537 million adults worldwide and is estimated to increase to 783 million by the year 2045 [3,4]. For instance, in South Africa, diabetes has steadily increased, especially among low-income communities [5]. Medicinal plants are widely known to complement conventional medicines, and their utilization is linked to various cultural and community groups, particularly those that demonstrate efficacy in the treatment of chronic diseases such as diabetes [6,7]. Several studies have documented the strength of medicinal plants in treating and managing various diseases [8,9,10]. However, several limitations have been identified, as they often present long-term side effects, toxicity, and unknown adverse effects, thus impeding their wider acceptability [11,12,13]. Yet, medicinal plants contain several different biologically active compounds that provide healthy benefits [14,15,16]. The World Health Organisation (WHO) states that about 70–80% of the global population still relies solely on medicinal plants for healthcare management, promoting the sustainable health and well-being of individuals [6]. Since time immemorial, medicinal plants have been involved in a plethora of complex biochemical, metabolic, and physiological mechanisms [10]. Several studies have identified that many rural communities in South Africa still rely on medicinal plants to manage diabetes because they are highly trusted among communities and affordable [10,16,17,18,19,20]. Therefore, scientific investigation of medicinal plants will continue to be a veritable and valuable source of ethnobotanical knowledge, preserving cultural diversity, facilitating co-integration into evidence-based healthcare and contributing to drug discovery and development. In this review, we documented the phytochemical, pharmacological, and toxicological profiles of *Silybum marianum* and *Brachylaena discolor* in managing type 2 diabetes. The study showed that both plants offered several promising potential benefits that could be explored when combined as an herbal formulation and consumed as an add-on dietary vegetable for people living with type 2 diabetes.

### 1.1. Asteraceae Family in the Management of T2DM

*Silybum marianum* and *Brachylaena discolor* belong to the Asteraceae family and are taxonomical groups that comprise approximately 32,000 plant species and are unique for their flowering characteristics [21,22,23]. Several ethnobotanical studies have reported that the Asteraceae family has nutritional and medicinal properties and is often used for food and medicinal purposes [18,21,22,23,24]. Some of the plant species in this family include chamomile, marigolds, sunflowers, dandelions, *Silybum marianum* (thistles), and *Brachylaena discolor* (coast silver oak), among others. They are rich in medicinal components and are highly nutritionally functional foods when consumed [25,26,27,28]. Many plant species in Asteraceae have been investigated for their pharmacological potential, including their antioxidant, anti-inflammatory, antimicrobial, and cytotoxic effects due to the presence of multiple secondary metabolites [29,30]. A hallmark phytochemical group within this family is the sesquiterpene lactones, which are significant for their bitter characteristic, an organoleptic diagnostic feature that is recognized for their significant role in modulating various biochemical and cellular pathways [25,31,32].

### 1.2. Origin, Morphology, and Biology of Silybum marianum

**Figure 1:**
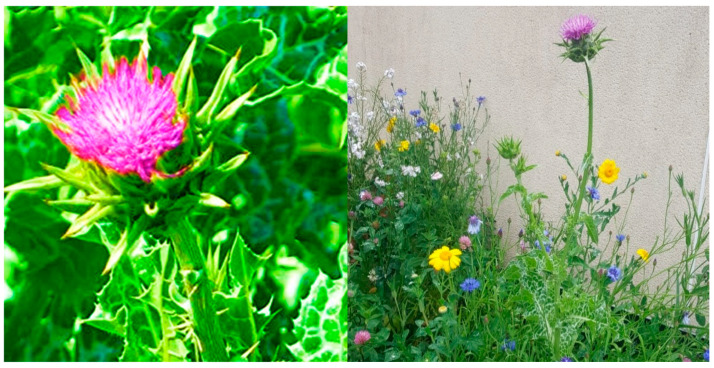
Morphology of *S. marianum*.

*Silybum marianum* (L.) Garten, commonly known as milk thistle, is a spiny herbaceous plant that belongs to the family of Asteraceae [33,34]. *Silybum marianum* is originally native to Mediterranean areas, Asia, Southern Europe, Australia, South America, Africa, and some parts of Russia, and it migrated to other countries worldwide due to its medicinal properties [35]. *Silybum marianum* cultivation varies based on different sowing times [36]. For instance, it is a biennial crop when it grows in the wild and might be regarded as an annual crop when cultivated [36]. The seeds germinate in autumn and flower in summer, reaching a vegetative cycle of 8–9 months when grown in the wild [37]. The morphological features of the plants show a glabrous or slightly downy stem that is erect and branched in the upper part [38]. The young leaves of *S. marianum* are tender, juicy, crisp, and refreshing, thus making it a tender vegetable for consumption [39]. The stem leaves are smaller than those of the rosette, between 50 and 60 cm in length and 20 and 30 cm in width, and their leaves are broad, lanceolate, and include purple tubular petals (Figure 1). Their height ranges between 40 and 200 cm and the leaves show notable white veins along the top side as a unique feature that aids in taxonomical identification [40].

### 1.3. Ethnopharmacological Utilization of S. marianum

All parts of *S. marianum*, including the roots, aerial parts, leaves, and seeds, are used for medicinal purposes, such as treating liver disorders and gallbladder disease, protecting from snake bites, cardiac disorders, fevers, rheumatism, liver cirrhosis, and controlling blood sugar levels and gastroenteritis [33,38,41]. The seeds, roots, bark, leaves and immature fruits have been used to treat gastrointestinal, type 2 diabetes, and diarrhea, while the leaves are applied to sores and manage hemorrhoid pain [36].

### 1.4. Chemical Components of Silybum marianum and Their Role in Anti-Diabetic Activity

**Figure 2:**
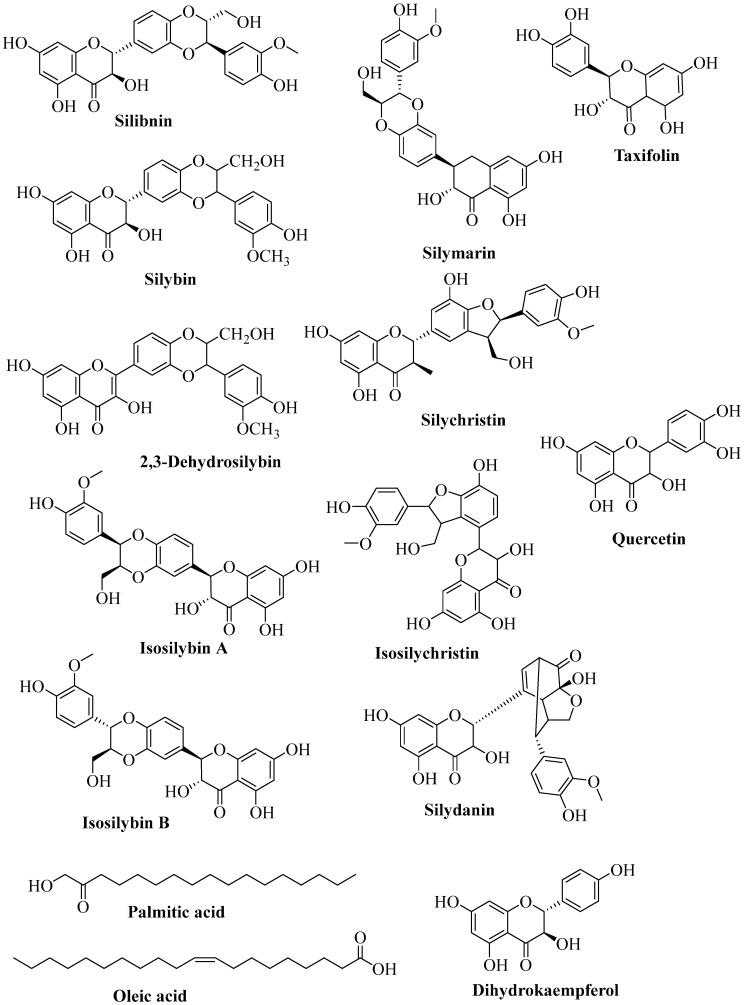
Chemical components of *S. marianum*.

Several chemical compounds have been isolated from *S. marianum*, which include silymarin, silibinin, isosilychristin, isosilybin A and isosilybin B, silybin A and silybin B, silychristin, silydianin and taxifolin, apigenin 7-*O*-*β*-(2″-*O*-*α*-rhamnosyl) galacturonide, kaempferol 3-*O*-*α*-rhamnoside-7-*O*-*β*-galacturonide, apigenin 7-*O*-*β*-glucuronide, apigenin 7-*O*-*β*-glucoside, apigenin 7-*O-β*-galactoside, kaempferol-3-*O*-*α*-rhamnoside, kaempferol and quercetin [42,43,44], linoleic (35–55%), oleic (24–30%) acids, together with palmitic (8–12%), behenic (3–9%), and other fatty acids and oils (Figure 2 and Table 1). *Silybum marianum* produces edible oils, protein powder, and forage. It exhibits excellent antioxidant effects, making it very suitable for use in cosmetics to protect the skin [38]. Specifically, silymarin, a major component in the plant, possesses free radical scavenging properties and inhibits lipid peroxidation. It was noted that these properties are likely due to the presence of the *β*-ring catechol group as well as the donation of hydrogen electrons, which prevents the depletion of free radicals [38,45,46]. Silibinin also showed significant protection by improving the level of superoxide dismutase and reducing the high triglyceride levels in the hepatic system, thereby impeding oxidative damage [47]. Awla et al. [48] reported that silydianin and silychristin in the extract of *S. marianum* reduced serum triglyceride levels and decreased glucose levels significantly (*p* < 0.001). These compounds prevented weight gain and normalized blood pressure in a high-fat/high-fructose diet compared to the control group [49]. The oils from *S. marianum* have the potential to improve the nutritional value of food by providing additional vitamins, proteins, and linoleic acid, as well as low-fat content and low cholesterol levels [38,50,51].

### 1.5. Toxicity

*Silybum marianum* is considered safe for both human and veterinary use when administered in liquid or solid dosage forms. Clinical studies have reported that silymarin is well tolerated at 700–1500 mg three times daily for 24 weeks [52]. According to the European Medicines Agency (EMA), some side effects of *S. marianum* include dry mouth, nausea, upset stomach, stomach irritation, diarrhea, headache, and allergic reactions (including skin inflammation, itching, rash, sudden severe allergic reaction, and asthma [53]. The ethylacetate extract of *S. marianum* showed normal neurological, behavioral, and autonomic profiles at 2000 mg/kg, and no mortality was recorded during the 48 h of oral administration [48]. Other compounds, such as silybin, silydianin, and silychristin, showed no cytotoxic and genotoxic effects at a 100 μM concentration. It was found that silymarin showed mutagenic activity in *S. typhii* strains in the presence of metabolic enzymes [52,54]. However, some gastrointestinal symptoms might occur, which include nausea and laxative effects, due to increased bile secretion and diarrhea [52]. *S. marianum* is affected by some mycotoxin-based dietary supplements, which are toxigenic and have the potential to reduce the beneficial effect of silymarin when consumed [53]. Many European countries, such as Austria, Belgium, Croatia, the Czech Republic, Estonia, France, and Germany, have established the use of *Silybum marianum* capsules in tablets and pharmacopeia standards [55]. Comprehensive toxicological studies will be helpful to understand pathways and multi-target disease conditions in our future studies. Maaliah et al. reported that the extract of *S. marianum* is hepatotoxic and could lead to acute and chronic liver diseases [56]. Kazazis et al. [57] stated that silymarin is safe at 13 g/day and could be tolerated in chemotherapeutic agents. However, it was suggested that doses as high as 20 g/day could cause asymptomatic liver toxicity [57]. In an in silico study, it was evaluated that the extract of *S. marianum* showed high absorption, favorable metabolism, and minimal toxicity with no hERG channel inhibition or hepatotoxicity [58]. Soleimani et al. suggested that silymarin is considered safe during pregnancy [52]. However, there is a need for caution when taken during pregnancy, as it might interfere with other drugs, leading to drug–drug interactions. Rysava et al. [59] reported that silymarin and its polyphenols affect the Nrf2 signaling pathway in human skin cells, but their effect after repeated long-term application to the whole skin was not ascertained. Silibinin, another potential chemical component, increased the expression of glucagon-like peptide-1 receptor (GLP1R) in the duodenum, and the activation of neurons in the nucleus of the solitary tract decreased hepatic glucose production at 100–300 mg/kg in rats when fed with a high-fat diet, alongside alleviating streptozotocin-induced diabetes [60]. The in vivo activity of silibinin alleviated non-alcoholic fatty liver diseases and insulin resistance by modulating the IRS-1/PI3K/Akt pathway [61]. In addition, silymarin has been reported to ameliorate insulin resistance, dyslipidemia, and inflammation and reconstitute the bile acid pool in the liver of experimental animals with diet-induced obesity [62]. Also, it was noted that silymarin showed significant overall health performance on the lens even in type 1 diabetic experimental rats [63]. Wang et al. stated that silymarin reduced liver and pancreatic protein damage and creatinine levels in some animal studies [64,65,66]. Silymarin supplementation reduced fasting blood sugar, serum insulin, and homeostasis, and there was a significant increase in high-density lipoprotein cholesterol levels and the quantitative insulin sensitivity check index [67]. Tuorkey and colleagues reported that silymarin extracted from the seeds significantly normalized the enzyme levels and decreased the serum glucose level, cholesterol, and triglycerides compared to the negative control group [68]. Silymarin enhanced the antioxidant capacity and normalized liver and renal function enzymes [68].

### 1.6. Anti-Diabetic Activity of S. marianum

*Silybum marianum* has received considerable scientific attention due to its anti-diabetic, antioxidant, and anti-inflammatory properties [69]. The oral administration of *S*. *marianum* extract to alloxan-induced diabetic rats reduced their MDA levels and validated its antioxidant properties. For instance, at 75 mg/kg, there was a significant decrease in MDA levels and lipid peroxidation and the half-maximal inhibitory value was found to be 13.88 ± 0.25 mg/kg. Methanolic leaf extracts of *S. marianum* demonstrated significant reducing power for some metabolic enzymes, with *β*-carotene bleaching inhibition at doses as low as 0.02 mg/mL in lipid peroxidation assays [70]. *Silybum marianum* extract at 400 mg/kg decreased the blood glucose levels significantly (*p* < 0.05) and revealed a significant effect on the cholesterol, liver enzymes, and kidney functions of diabetic rats compared with those that received 200 mg/kg [71]. The findings above suggest dose-dependent therapeutic potential, and higher doses show better efficacy of the extract. Furthermore, in an experimental animal study on the extract, there was a significant improvement in some of the pancreatic enzymes and plasma glutathione, which thus confirmed protection against lipid peroxidation and enhanced plasma glucose induced by alloxan in mice [72,73]. Maaliah et al. [56] reported that the ethanol extract of *S. marianum* decreased serum glucose, triglyceride, total cholesterol, low-density lipoprotein, very-low-density lipoprotein, and malondialdehyde (MDA) levels. The methanol extract demonstrated an inhibitory concentration, IC_50_, of 5.2 ± 0.07 μg/mL [74]. The aqueous extract of *S. marianum* from the aerial parts revealed a decrease in blood glucose levels [75]. In an in vivo study conducted by Villiger and co-authors on ethanol fruit extract [76], it was observed that 80% of *α*-glucosidase inhibitory activity was reported at 100 μg/mL compared to that of acarbose (1–1500 μM). Some major chemical components of *S. marianum* are used as a single therapy to lower blood glucose levels; however, when combined with other drugs such as phosphatidylcholine, simvastatin, and vitamin E, they can lower blood glucose even more significantly [77,78,79]. The hypoglycemic and antihyperlipidemic effects of the extract were beneficial in BSA glycation and *α*-amylase activity studies [56]. Its antioxidant activity has been extensively studied, alongside how it contributes to the antioxidant potential [80,81]. *Silybum marianum* displays potent antioxidant capabilities, which are principally attributed to the presence of polyphenolic components, particularly flavonolignans such as silymarin and silibinin [81]. The extract of *S. marianum* possessed a strong antioxidant profile, measured using a microwave-enhanced technique, and the extract contained 251.2 ± 1.2 mg GAE/g of total phenolics and 125.1 ± 1.6 mg QE/g of total flavonoids, while strong antioxidant activity was observed through half-maximal inhibitory concentration (IC_50_) values of 19.2 ± 2.3 μg/mL for DPPH, 7.2 ± 1.7 μg/mL (ABTS), 22.2 ± 1.2 μg/mL (CUPRAC), 35.2 ± 1.8 μg/mL (Phenanthroline), and 24.1 ± 1.2 μg/mL (FRAP) [58]. The anti-diabetic effects were significant at 18.1 ± 1.7 μg/mL (*α*-glucosidase) and 26.5 ± 1.3 μg/mL (*α*-amylase), respectively [58]. The aerial part of *S. marianum* showed significant free radical scavenging and ferric-reducing antioxidant power (FRAP), with an IC_50_ value of 1.73 mg/mL. In contrast, the methanolic extracts obtained during the flowering stage showed the highest activity in both DPPH and ABTS assays, with IC_50_ values ranging from 3.45 to 4.08 mmol compared with Trolox equivalents per 100 g dry weight [82]. Some of the isolated compounds have been reported to reduce oxidative stress, which is significant in the management of type 2 diabetes [83]. Co-morbid diseases contribute to excessive ROS formation and worsen diabetic conditions when not controlled [84]. Thus, silymarin reduced oxidative stress and inflammation in streptozotocin-induced diabetes mice [85].

In a synergistic study conducted by Nasir et al. [86] on dilute acetic acid extracts of *N. sativa* and *S. marianum* seeds, they showed the potential of various fractions to have different inhibitory *α*-amylase activities. Moreover, subfraction (F5) demonstrated the strongest inhibitory *α*-amylase activity due to the presence of various phytochemical components. These findings also revealed that when anti-diabetic medicinal plants are combined in herbal formulations, they significantly decrease blood glucose levels, increase pancreatic secretions, and maintain good antioxidant status with little or no side effects in the body tissues or systems. The seeds were found to be richer in antioxidants than other parts of the plant and a rich source of protein, potassium, calcium, and magnesium [32]. In a clinical trial conducted by Wang et al., some of the health benefits of phytochemical compounds found in *S. marianum* showed that patients who received silymarin extract had a significant improvement in their glycemic indices, lipid profiles, and antioxidant indices, while the hs-CRP levels in patients decreased [67]. Huseini et al. reported the effect of antioxidant nutrients on the glycemic control of diabetic patients in experimental and clinical studies [20]. It was stated that the average fasting blood glucose level in the silymarin group decreased significantly (*p* < 0.001) after 4 months of silymarin treatment, while the average fasting blood glucose level in the placebo group at the beginning of the study was increased significantly (*p* < 0.0001) after 4 months of placebo treatment [87]. The isolation of mariamides A and mariamides B revealed the potential of the compounds to regulate the insulin signaling pathway by increasing protein tyrosine phosphatase 1B (PTP1B) inhibition [88]. Silybin (A and B) acted as a non-competitive PTP1B inhibitor, and IC_50_ values of 1.54–1.37 μM were recorded [89]. Silibinin exhibited a hyperglycemia-reducing effect and decreased the elevation of ALT, which prevented the decrease in insulin in a streptozotocin (STZ)-induced diabetic rat model [42]. In a study conducted on obese db/db mice, silibinin reduced insulin resistance and prevented myocardial and hepatic damage [90]. Silibinin significantly suppressed H_2_O_2_ production in rat macrophages in a dose-dependent manner and outperformed standard antioxidant control at comparable concentrations. Further analyses showed strong antioxidant potential in ethanolic and aqueous extracts of *S. marianum* (IC_50_ = 36 and 44 µg/mL, respectively) [91]. It was noted that silibinin inhibited hIAPP fibrillization by suppressing the toxic oligomerization of hIAPP and enhancing the viability of pancreatic β-cells; therefore, silibinin may serve as a potential therapeutic agent for T2DM [92]. Other pharmacological activities also include hepatoprotectivity against ischemia/reperfusion injury, renal protectivity, cardioprotectivity, neuroprotectivity, and antinociceptive, analgesic, antimicrobial, anti-ulcerative, and immunomodulatory effects [30,93,94,95].

**Table 1:** Phytochemical components present in *S. marianum*.

Plant Sources	Compound Class	Compound Name	Molecular Weight (g/mol)	Chemical Formula	CAS Number	Biological Activity	References
	Flavonolignans						
Seeds Leaves Root		silymarin	482.4	C_25_H_22_O_10_	22888-70-6	anti-diabetic, anti-inflammatory properties, anti-diarrhea, and antioxidant activity.	[27,46]
Seeds Leaves Root		silybin A	482.4	C_25_H_22_O_10_	22888-70-6	antioxidant, anti-inflammatory, anti-fibrotic, hepatoprotective, and anti-diabetic activity.	[52,54]
Seeds Leaves Root		silybin B	482.4	C_25_H_22_O_10_	142797-34-0	antioxidant, anti-inflammatory, anti-fibrotic, hepatoprotective, and anti-diabetic activity.	[52,54]
Seeds Leaves Root		silydianin	482.4	C_25_H_22_O_10_	29782-68-1	antioxidant, anti-inflammatory, anti-fibrotic, hepatoprotective, and anti-diabetic activity.	[64,85]
Seeds Leaves Root		isosilybin A	482.4	C_25_H_22_O_10_	142796-21-2	antioxidant, anti-inflammatory, anti-fibrotic, hepatoprotective, and anti-diabetic activity.	[52,54]
Seeds Leaves Root		isosilybin B	482.4	C_25_H_22_O_10_	142796-22-3	antioxidant, anti-inflammatory, anti-fibrotic, hepatoprotective, and anti-diabetic activity.	[52,54]
Seeds Leaves Root		silychristin	482.4	C_25_H_22_O_10_	33889-69-9	antioxidant, anti-inflammatory, anti-fibrotic, hepatoprotective effects, and anti-diabetic activity	[89,96]
stem		2,3-dehydrosilybin	480	C_25_H_22_O_10_	25166-14-7	shows potential cardioprotective effects, strong antioxidant, anticancer, anti-lipid peroxidation, and cell damage attenuation properties.	[97,98]
	Phenylpropanoids						
Seeds Leaves Root		mariamide A	756.3	C_42_H_46_N_4_O_10_	Not known	antioxidant and anti-diabetic activities.	[88]
Seeds Leaves Root		mariamide B	407.2	C_21_H_24_N_2_O_5_	Not known	antioxidant and anti-diabetic activities.	[88]
	Flavonoids						
Seeds		3, 3′, 5, 5′, 7-pentahydroxyflavanone	305.1	C_15_H_12_O_7_	215257-15-1	anticancer and antioxidant activities	[99]
Stem		taxifolin	304.3	C_15_H_12_O_7_	480-18-2	antioxidant, anti-inflammatory, and anticancer effects, neuroprotective activity, benefits for Alzheimer’s disease, cardioprotectivity, anti-diabetic activity by improving insulin sensitivity, and liver protection.	[100]
Seeds Leaves Root		quercetin	302.23	C_15_H_10_O_7_	117-39-5	antioxidant, anti-inflammatory, anti-fibrotic, hepatoprotective effects and anti-diabetic, antioxidant, anti-inflammatory, anti-fibrotic, hepatoprotective and anti-diabetic activities.	[42,43,44]
Seeds Leaves Root		kaempferol	286.24	C_15_H_10_O_6_	520-18-3	antioxidant, anti-inflammatory, anti-fibrotic, hepatoprotective effects and anti-diabetic activity.	[42,43,44]
	Flavonoid glycosides						
Seeds Leaves Root		apigenin 7-*O*-*β*-(2″-*O*-*α*-rhamnosyl) galacturonide	592.5	C_27_H_28_O_15_	124167-97-1	anti-inflammatory and antioxidant effects, cardiovascular disease management, neuroprotection, and anti-diabetic properties by reducing oxidative stress and insulin resistance.	[101]
Seeds Leaves Root		kaempferol 3-*O*-*α*-rhamnoside-7-*O*-*β*-galacturonide	608.1	C_27_H_30_O_16_	124167-98-2	antioxidant, anti-inflammatory, and potential anticancer activity.	[27,46]
Seeds Leaves Root		apigenin 7-*O*-*β*-glucuronide	446.36	C_21_H_18_O_11_	29741-09-1	anti-diabetic and antioxidant activities	[42,43,44]
Seeds Leaves Root		apigenin 7-O-*β*-galactoside	432.4	C_21_H_20_O_10_	578-74-5	antioxidant, anti-inflammatory, anti-fibrotic, hepatoprotective effects, and anti-diabetic activity.	[42,43,44]
Seeds Leaves Root		kaempferol-3-*O*-*α*-rhamnoside	432.4	C_21_H_20_O_10_	482-39-3	antioxidant, anti-inflammatory, anti-fibrotic, hepatoprotective effects, and anti-diabetic activity	[42,43,44]
	Fatty acids						
Seeds		oleic acid	282.5	C_18_H_34_O_2_	112-80-1	anti-inflammatory and antioxidant effects, and cardiovascular disease and obesity treatment	[42,43,44]
Seeds		linoleic acid	280.4	C_18_H_32_O_2_	60-33-3	potential anti-neoplastic and pro-apoptotic effects against certain cancers and anti-inflammatory activity	[42,43,44]
Seeds		palmitic acid	256.4	C_16_H_32_O_2_	57-10-3	anti-inflammatory, anticancer, and anti-viral agent by modulating immune signaling pathways like NF-κB and inducing apoptosis in cancer cells	[42,43,44]

### 1.7. Origin, Morphology, and Biology of Brachylaena discolor

**Figure 3:**
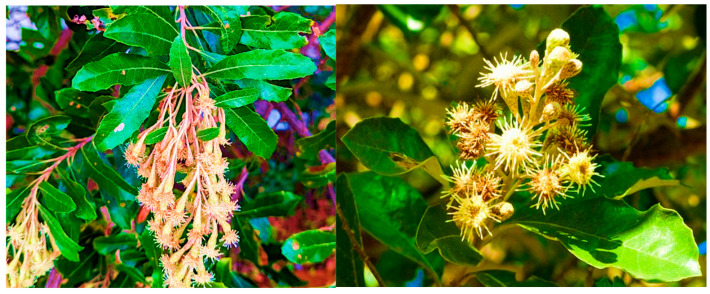
Morphology of *Brachylaena discolor*.

*Brachylaena discolor* DC. is popularly known as the coast silver oak. This plant is a shrubby plant indigenous to South Africa and belongs to the family Asteraceae [102]. The Genus name *Brachylaena* is derived from the Greek brachys (short) and klaina (cloak), referring to the disproportion between the relatively long florets and the shorter involucral bracts [10,103]. The specific epithet discolor reflects the bicolor foliage, which is dark greenish on the adaxial (upper) surface and pale, silver-gray on the abaxial (lower) surface (Figure 3). This feature informs the species’ common name “coastal silver oak” [103]. The plant is variably evergreen or deciduous and may attain a height of up to 30 m [104,105]. Its bark ranges in coloration from dark gray to reddish-brown and is lenticellate and fissured, while the branches are characteristically rough. The leaves are lanceolate to ovate, with entire or slightly serrated margins, and display dual coloration. Inflorescences are borne in axillary panicles, comprising numerous creamy-white florets. The fruit is a cypsela (achene) and is capped by a pappus of cream to brown bristles, facilitating anemochory [106]. The plant generally prefers dunes and coastal places as habitats and can grow to about 10 m high, depending on environmental factors, so it might be a bushy shrub or a small tree of about 10 m high [107]. Among Afrikaans people, it is called *Kusvaalbos*, *Muakawura*, and *Mupasa* in *Shona* and *Iphahla/Umpahla* in *IsiZulu* in Southern Africa. *Brachylaena discolor* is distributed across Southern Africa, including Zimbabwe, Botswana, Eswatini, Zambia, Mozambique, and South Africa. It occurs in varied ecological niches such as termite mounds, sandy soils, secondary woodlands, evergreen forests, forest margins, rocky slopes, and hillsides, at elevations ranging from sea level to approximately 1900 m above sea level [105,108].

### 1.8. Ethnopharmacological Utilization of Brachylaena discolor

*Brachylaena discolor* is widely used among traditional healers across Southern African countries in Botswana, Eswatini, Mozambique, South Africa, Zambia, and Zimbabwe [19]. The roots and leaves are frequently traded in herbal markets within KwaZulu-Natal and Gauteng provinces of South Africa [109]. Traditional remedies use aqueous extractions (infusions or decoctions) of the plant twigs, stems, bark, roots, or leaves. For instance, the leaves are boiled and the infusion is taken orally [110]. *B. discolor* is commonly mixed with other herbs to treat many illnesses [111]. Several ethnomedicinal uses include anti-helmintics, general tonics, treatments for ailments such as female infertility; dermatological conditions; renal disorders; anticancer, anthelmintic, and antihyperglycemic effects; gastrointestinal disturbances; and respiratory tract infections [112]. In South Africa, *Brachylaena discolor* is among the species commonly used to manage diabetes, alongside *B. ilicifolia* and *B. elliptica* [113,114,115]. Topical preparations incorporating *B. discolor* twigs with species such as *Euphorbia tirucalli*, *Hypoxis hemerocallidea*, *Ozoroa engleri*, and *Senecio serratuloides* are traditionally used to manage sores and cutaneous infections, thus making it ideal to treat diabetes [115]. Ethnopharmacological studies revealed that these compounds contribute to other biological activities such as antibacterial, cytotoxic, antifungal, and anti-leishmanial effects [16].

### 1.9. Chemical Components of Brachylaena discolor and Their Role in Anti-Diabetic Activity

**Figure 4:**
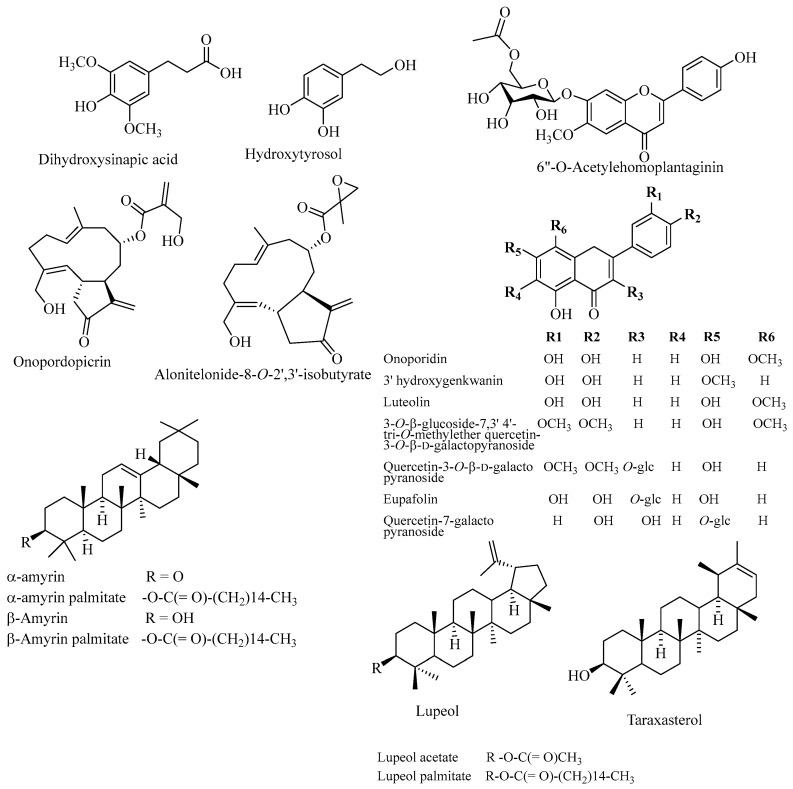
Chemical components from *Brachylaena discolor*.

Several chemical compounds have been identified from *B. discolor*, including alkaloids, flavonoids, phenolic acids, phlobotannins, saponins, sesquiterpene lactones, steroids, tannins and terpenoids, *α*-amyrin acetate, *β*-amyrin acetate, ψ-taraxasterol acetate, taraxasterol acetate, lupeol, *α*-amyrin palmitate, *β*-amyrin palmitate and lupeol palmitate, *α*-amyrin, *β*-amyrin, and taraxasterol, as shown in Figure 4 and Table 2 [103]. Other reported isolated compounds include lupeol acetate, *β*-sitosteryl linolenate, *α*-tocopherol, genkwanin 5-*O*-*β*-D-glucopyranoside, onopordopicrin, and its epoxide derivatives such as alonitelonide-8-*O*-2′,3′-isobutyrate, hydroxytyrosol, dihydroxysinapic acid, 6″-*O*-acetyl 3′-hydroxygenkwanin, 6″-*O*-acetyl-homoplantaginin, dihydroxysinapic acid, quercetin3-*O*-glucoside-7, 3′, 4′-trimethyl ether, onoporidin, quercetin-7-galactopyranoside, luteolin, homoplantaginin, onoporidin, 3′-hydroxygenkwanin, luteolin, quercetin 3-*O*-glucoside-7, 3′, 4′-trimethyl ether, quercetin 3-*O*-*β*-D-galactopyranoside, eupafolin, quercetin-7-galactopyranoside-15-Dihydrodehydrozaluzanin C, 3-acetoxy-12-lupene, dehydrobrachylaenolide, di-hydrodehydrocostuslactone, brachylaenolide, dehydrocostuslactone, germacrene D, costunolide, dehydrozaluzanin C, furanoheliangolide, germacranolide, genkwanin-5-*O*-D-glucopyranoside, germacronolide epoxide, hydroxytyrosol, lupeol acetate, onopordopicrin, salonitenolide, 9-oxo-nerolidol, salonitenolide-8-*O*-2,3-epoxy-isobutyrate, and linolenic acid [103,114,116,117,118]. It was stated that the daily administration of *β*-amyrin palmitate showed strong anti-diabetic activity in alloxan-induced diabetic and streptozotocin-induced diabetic rats at 50 mg/kg body weight [119]. *β*-amyrin showed significant protection of *β*-cell integrity in streptozotocin-challenged mice [120]. Xu et al. [121] also found that *β*-amyrin improved kidney injury in diabetic nephropathy mice and suppressed the inflammatory response and apoptosis of HG-stimulated HK-2 cells. The oral administration of *α*- and *β*-amyrins compounds showed significant reductions in blood glucose levels, total cholesterol, and serum triglyceride [122]. It was stated that at a dose of 100 mg/kg, there was a significant reduction in blood glucose and a strong lipid-lowering effect [122]. These findings revealed the potential of *α*- and *β*-amyrins to enhance glucose metabolism and preserve pancreatic functions. *β*-amyrin also has significant antibacterial and anti-diabetic properties through its enzymatic inhibitory activity, notably achieving an inhibition rate of between 49.8 ± 0.3% and 69.3 ± 1.0% at a concentration of 10 µg/mL in *α*-amylase inhibitory activity studies [123]. It was reported that *β*-amyrin at doses of 10 and 50 mg/kg did not exhibit a significant decrease in food and water consumption in 4 weeks of treatment. Still, noticeable signs of toxicity were observed at doses greater than 30 mg/kg administered orally. The toxicity signs included tremor, ataxia, increased respiration, and decreased activity [124]. *β*-amyrin palmitate possibly blocked the entry of glucose from the intestine. This resulted in a significant decrease (*p* < 0.001) in the non-fasting levels of blood glucose without interfering with the body weight and liver weight. From our findings, toxicological information on *B. discolor* was limited, and the extract exhibited activities with a half-maximal lethal dose (LD_50_) value of 0.004 mg/mL [125].

### 1.10. Anti-Diabetic Activity of Brachylaena discolor

*Brachylaena discolor* is a major plant used in managing type 2 diabetes in the Eastern Cape of South Africa [112]. The biological relevance of the crude extract was reported using dichloromethane–methanol extraction for its ability to inhibit *α*-glucosidase activity [126,127,128]. The findings demonstrated that the extract showed superior inhibitory activity compared to acarbose, a standard pharmaceutical agent. However, attempts to evaluate the enzyme inhibitory potential of the isolated pure compounds were unsuccessful due to their poor solubility in the assay medium [128]. Given the diverse traditional applications and promising preliminary pharmacological evidence, further chemical studies should focus on elucidating other active components of *B. discolor* (Table 2). The effects of organic and aqueous extracts derived from the leaves, roots, and stems of *B. discolor* on glucose uptake in 3T3-L1 adipocytes and liver cells improved glucose utilization across many cell lines, thus indicating promising anti-diabetic activity [129]. The methanolic leaf extracts of *B. discolor* administered to streptozotocin-induced diabetic rats at 50 mg/kg and 150 mg/kg resulted in a significant reduction in blood glucose levels [113]. Further alterations in body weight and key biochemical parameters such as total bilirubin, creatinine, and alkaline phosphatase confirmed the potential of the extract to have anti-diabetic efficacy. The inhibitory effects of aqueous and methanolic leaf extracts on the digestive enzymes *α*-amylase and *α*-glucosidase can be used to manage postprandial hyperglycemia. In a study conducted by Mellem and colleagues [126] on the antioxidant activity of aqueous and methanolic extracts, it was found that methanol and aqueous extracts exhibit IC_50_ values of 92.3 μg/mL and 82.8 μg/mL, respectively, and a moderate free radical scavenging activity was shown [126]. The *B. discolor* extracts demonstrated notable inhibitory activity, with IC_50_ values ranging from 1.8 to 11.0 mg/mL, in comparison to the control, with IC_50_ values of 0.03–1.2 mg/mL [113]. Adamu et al. reported that the acetone extracts revealed moderate antioxidant capacity, with a Trolox Equivalent Antioxidant Capacity (TEAC) value of 0.2 in the ABTS assay and an EC_50_ of 2.6 mg/mL in the DPPH assay. Dikhoba et al. further stated the effects of the acetone leaf extract of *B. discolor* on ABTS and DPPH, and IC_50_ values of 0.03 mg/mL and 0.2 mg/mL were reported, benchmarked against ascorbic acid (0.5 mg/mL). As a result, it was found that *B. discolor* is a rich source of antioxidant compounds [127]. However, it was stated that the *Brachylaena discolor* plant is still underutilized in managing diabetes and that the anti-diabetic potential of *Brachylaena discolor* should be elucidated to uncover the active ingredients and other pharmacological activities [20,129].

**Table 2:** Phytochemical components present in *Brachylaena discolor*.

Plant Sources	Compound Class	Compound Name	Molecular Weight (g/mol)	Chemical Formula	CAS Number	Biological Activity	References
	Triterpene						
Stems Fruits Leaves		α-amyrin	456.7	C_30_H_48_O_3_	638-95-9	enhances periodontal inflammation, anti-diabetic effects, neutrophil infiltration, and oxidative stress management	[130,131]
Stems Fruits Leaves		*β*-amyrin	426.0	C_30_H_50_O	559-70-6	anti-inflammatory, antibacterial, antinociceptive (pain-relieving), hepatoprotective (liver-protective), and anti-diabetic effects, neuroprotective agent, antifungal agent, and antioxidant properties.	[131]
Stems Fruits Leaves		*α*-amyrin palmitate	665.1	C_46_H_80_O_2_	22255-10-3	anti-inflammatory activity, improves brain neuronal hormones, anti-diabetic activity, neutrophil infiltration, and helps in managing oxidative stress	[131]
Stems Fruits Leaves		*β*-amyrin palmitate	665.1	C_46_H_80_O_2_	5973-06-8	antidepressant, hypoactive, anti-diabetic, and hypolipidemic activities	[122]
Stems Fruits Leaves		α-amyrin acetate	468.0	C_32_H_52_O_2_	863-76-3.	anti-inflammatory, antihyperglycemic, hepatoprotective, and antifungal effects	[131]
Stems Fruits Leaves		ψ-taraxasterol acetate	469.4	C_32_H_53_O_2_	4586-65-6	neuroprotective, anticancer, and anti-diabetic properties, inhibiting pro-inflammatory cytokines and reducing oxidative stress	[132,133]
Stems Fruits Leaves		taraxasterol acetate	468.8	C_32_H_52_O_2_	6426-43-3	anti-inflammatory, anticancer, antioxidant, and neuroprotective activities.	[132,133]
Stems Fruits Leaves		taraxasterol	426.7	C_30_H_50_O	1059-14-9	anti-inflammatory, anticancer, antioxidant, and neuroprotective activities.	[132,133]
Stems Fruits Leaves		lupeol acetate	468	C_32_H_53_O_2_	1617-68-1	acts as an anti-inflammatory agent, has anti-diabetic activity, suppresses pro-inflammatory cytokines, has antioxidant and neutralizing anti-venom components and improves antioxidant status of cells.	[128]
Stems Fruits Leaves		lupeol palmitate	663.6	C_46_H_80_O_2_	32214-80-5	reduces blood glucose levels, improves antioxidant levels, and reduces inflammatory markers.	[134]
Stems Fruits Leaves		lupeol	409.4	C_30_H_49_	545-47-1	reduces blood glucose levels, improves antioxidant levels, and reduces inflammatory markers	[134]
Stems Fruits Leaves		luteolin	286.2	C_15_H_10_O_6_	491-70-3	improves glucose and lipid metabolism by reducing insulin resistance, enhancing glucose uptake, inhibiting inflammatory pathways, and scavenging free radicals.	[135]
	Sesquiterpene						
Leaves Stem Fruits		onopordopicrin	349.1651	C_19_H_24_O_6_	19889-00-0	antioxidant, anti-inflammatory, and cytotoxic activities.	[103,114,136]
	Organic acids						
Stems Fruits Leaves		dihydroxysinapic acid	180.2	C_9_H_8_O	331-39-5	potent antioxidant, anti-inflammatory, and anticancer properties, along with antiviral, anti-diabetic, antimicrobial, cardioprotective, immune-stimulatory, and neuroprotective effects.	[137,138]
Stems Fruits Leaves		salonitelonide-8-*O*-,3-isobutyrate	348.1570	C_19_H_24_O_6_	Not known	anti-leishmanicidal activity and treats stomach pain, tuberculosis, and diabetes.	[103,114,136]
	Flavonoids						
Stems Fruits Leaves		hydroxytyrosol	154.2	C_8_H_10_O_3_	10597-60-1	strong antioxidant and anti-inflammatory effects and neuroprotective, cardioprotective, and anticancer properties by inhibiting tumor cell growth, inducing apoptosis, and improving cardiovascular health markers.	[137,139]
Stems Fruits Leaves		eupafolin	316.3	C_16_H_12_O_7_	520-11-6	increases glucose metabolism and antioxidant and anti-inflammatory activity.	[140]
Stems Fruits Leaves		3′-hydroxygenkwanin	300.3	C_16_H_12_O_6_	20243-59-8	offers cardiovascular protection, inhibits cancer cell growth, and reverses drug-induced DNA damage repair inhibition in liver cancer cells	[141]
	Flavonoid glycosides						
Stems Fruits Leaves		quercetin 3-*O*-glucoside-7,3′,4′-trimethyl ether	622.5	C_27_H_30_O_17_	Not known	anti-diabetic, antibacterial, anti-inflammatory, and antioxidant effects	[142]
Stems Fruits Leaves		quercetin-3-*O*-*β*-D-galactopyranoside	464.4	C_21_H_20_O_12_	482-36-0	anti-diabetic, antibacterial, anti-inflammatory, and antioxidant effects	[143]
Stems Fruits Leaves		quercetin-7-galactopyranoside	464.4	C_21_H_20_O_12_	482-36-0	antioxidant, anti-inflammatory, anti-diabetic, and cytoprotective effects and manages oxidative stress.	[143]

## 2. Conclusions

This study documented the anti-diabetic potential of *Silybum marianum* and *Brachylaena discolor* from the Asteraceae family to manage type 2 diabetes mellitus. Knowledge of their ethnobotanical utilization justified their pharmacological investigation as anti-diabetic plants. Several chemical components isolated from these plants have been shown to have the potential to lower blood glucose levels in many experimental studies. However, toxicological information on their long-term effects is limited, and safe dosages are a major concern. Commercialization of these plants is encouraged to provide economic benefits, and they are recommended as an add-on vegetable to dietary meals to lower blood sugar levels.

## Data Availability

No new data were created or analyzed in this study. Data sharing is not applicable to this article.
